# Graphite Oxide Improves Adhesion and Water Resistance of Canola Protein–Graphite Oxide Hybrid Adhesive

**DOI:** 10.1038/s41598-017-11966-8

**Published:** 2017-09-14

**Authors:** Nandika Bandara, Yussef Esparza, Jianping Wu

**Affiliations:** Department of Agricultural, Food and Nutritional Science, University of Alberta, Edmonton, Canada

## Abstract

Protein derived adhesives are extensively explored as a replacement for synthetic ones, but suffers from weak adhesion and water resistance. Graphite oxide (GO) has been extensively used in nanocomposites, but not in adhesives applications. The objectives of this study were to prepare functionally improved protein adhesive by exfoliating GO with different oxidation levels, and to determine the effect of GO on adhesion mechanism. GO were prepared by oxidizing graphite for 0.5, 2, and 4 h (GO-A, GO-B and GO-C, respectively). Increasing oxidation time decreased C/O ratio; while the relative proportion of C-OH, and C = O groups initially increased up to 2 h of oxidation, but reduced upon further oxidation. Canola protein-GO hybrid adhesive (CPA-GO) was prepared by exfoliating GO at a level of 1% (w/w). GO significantly increased (*p* < *0.05*) adhesion; where GO-B addition showed the highest dry, and wet strength of 11.67 ± 1.00, and 4.85 ± 0.61 MPa, respectively. The improvements in adhesion was due to the improved exfoliation of GO, improved adhesive and cohesive interactions, increased hydrogen bonding, increased hydrophobic interactions and thermal stability of CPA-GO. GO, as we proposed for the first time is easier to process and cost-effective in preparing protein-based adhesives with significantly improved functionalities.

## Introduction

Due to increasing concerns over environmental and human health implications of synthetic adhesives, researchers are looking for green materials/biobased adhesives using sustainable and renewable polymers^1–4^. Proteins are one of the most studied renewable polymers for adhesive applications^5^. Canola is the farm-gate crop in Canada while its meal after oil extraction finds limited value-added applications other than feed and fertilizer uses; thus research on canola protein gains the momentum as an alternative polymer source for adhesive preparation^5, 6^. However, similar to other proteins, canola protein derived adhesives also suffered from weak water resistance and adhesion strength, which might limit their widespread applications^5, 7^. Therefore, improving water resistance and adhesion strength of canola protein-derived adhesives is essential to succeed as a competitive alternative over synthetic ones. Our previous study found that exfoliating nanomaterials at lower addition levels could significantly increase the adhesion strength and water resistance of canola protein; especially, graphite oxide (GO) and nano crystalline cellulose (NCC) showed superior improvement over other nanomaterials^8^. The dry, wet and soaked adhesion strength of canola protein adhesives was increased from 6.38 ± 0.84 MPa, 1.98 ± 0.22 MPa, and 5.65 ± 0.46 MPa in the pH control samples to 10.37 ± 1.63 MPa, 3.56 ± 0.57 MPa, and 7.66 ± 1.37 MPa, respectively, for the 1% NCC addition (w/w, NCC/protein), and to 8.14 ± 0.45 MPa, 3.25 ± 0.36 MPa, and 7.76 ± 0.53 MPa for the 1% GO (w/w,GO/protein) addition^8^.

Although NCC showed greater improvement than GO, NCC is more expensive than that of GO. Furthermore, GO shows excellent exfoliation properties in aqueous and organic solvents, as well as in different polymer matrixes due to hydrophilic nature of GO^9^. Previous studies on composite materials showed that the improvements in mechanical, thermal and electrical properties were directly related to the exfoliation properties of nanomaterials in polymer matrix^2, 10^. Therefore, it is essential to use a nanomaterial with better exfoliation properties for adhesive preparation to improve mechanical strength of the adhesive^2^.

Carbon based nanomaterials such as carbon nanotubes, graphene, graphite oxide and aerographite have been extensively studied recently in polymer and composite applications, mainly due to their excellent mechanical, thermal and conductive properties^11, 12^. First isolated in 2004, graphene consists of two dimensional sheets of carbon molecules bonded via sp^2^-bonds^13^. Pristine graphene has unique material properties such as extremely high Young’s modulus (∼1 TPa), fracture strength (∼130 GPa), thermal conductivity (∼5000 Wm^−1^K^−1^) and specific surface area (2630 m^2^g^−1^) compared to other carbon based materials^14, 15^. Graphite oxide (GO), an intermediary product in mass scale production of graphene, possess similar material properties to graphene^15^. GO represents advantages over graphene, mainly due to their simplicity of production through chemical methods, hydrophilic properties, and potential to convert into graphene or graphene oxide^15, 16^ either by chemical^17, 18^ or thermal^19^ reduction methods before or after exfoliating in the polymer matrix. In addition, GO can form liquid crystals^20^ and microscopic assembly of graphene once incorporated in polymer matrix^21^, which could help develop homogeneous polymer composite with improved mechanical properties^13, 22^. The presence of oxygen containing functional groups imparts GO excellent hydrophilic properties, facilitating exfoliation in a polymer matrix^23^. Hydrophilic nature of GO is a vital property in preparing GO exfoliated adhesives using the solution intercalation method.

GO has been extensively explored in developing advanced nano-composites in combination with different polymers such as poly (vinyl acetate)^24, 25^, chitosan^26^, natural rubber^27^, poly (methyl methacrylate) and epoxy^10, 23^. However, there is scanty information available in literature on GO based adhesives, except one study found in literature regarding applicability of graphene in adhesive preparation. Khan *et al*. (2013) reported that incorporation of 3% graphene (dissolved in tetrahydrofuran) into poly (vinyl acetate) (PVA) adhesives improved both tensile strength (from 0.3 MPa to 0.75 MPa) and shear strength (from 0.5 MPa to 2.2 MPa) at dry conditions^28^. It is well known that the functionality of GO largely depends on the level of its oxidation^9, 23, 29^; therefore, there is a need to study the effect of different GO oxidation levels on adhesion strength and water resistance. We hypothesized that adding GO with different oxidation levels will change adhesion strength and water resistance of canola protein derived adhesives. The objectives of this research were to prepare GO with different oxidation levels under various oxidation time, to determine the effect of GO with different oxidation levels on adhesion properties, and to explore the mechanism of GO in adhesion improvement.

In this study, GO with different oxidation levels were prepared by oxidizing graphite at different oxidation times. Prepared GO samples were exfoliated in canola protein to produce canola protein-graphite oxide (CPA-GO) hybrid wood adhesive. The effect of oxidation time on C/O ratio, surface functional groups, interlayer spacing, and thermal properties were characterized to identify their effect on GO dispersion in protein matrix, structural and thermal changes, adhesion strength and water resistance of CPA-GO.

## Results and Discussion

The functionality of GO depends largely on the methods of preparation and conditions used in the process such as oxidation time and amount of oxidizer^23, 29^. In composite research, tailoring conditions of GO preparation have proven to change material properties such as flexural strength and conductivity^29^. However, to best of our knowledge, there were no previous reports in the literature regarding the effect of GO on adhesives derived from biobased polymers/protein-based polymers.

### Adhesion strength of canola protein-GO hybrid adhesives

**Figure 1:**
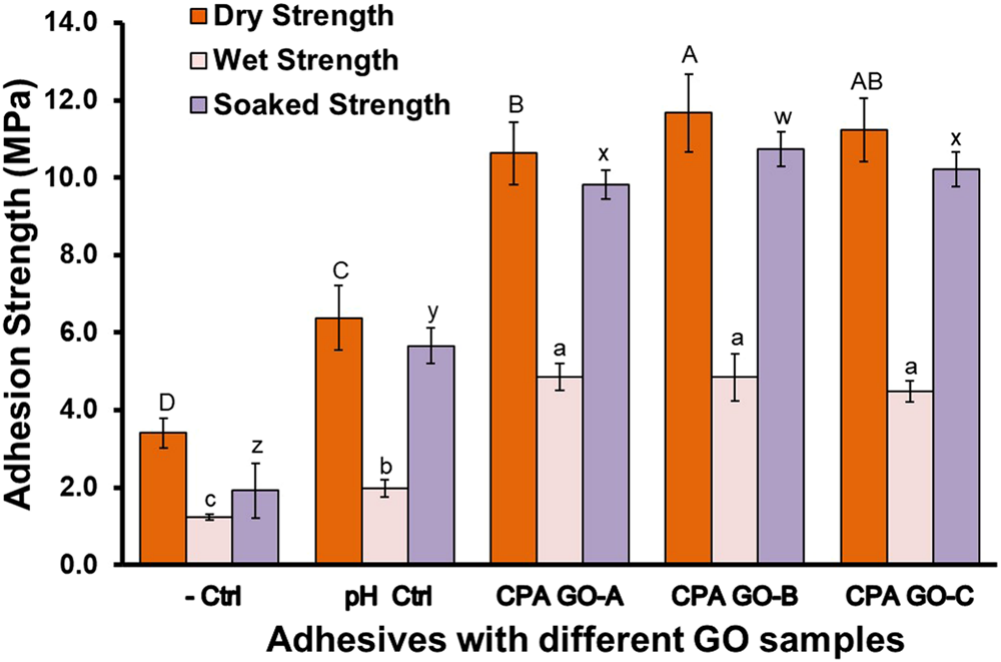
Adhesion strength of canola protein-graphite oxide hybrid wood adhesives prepared by exfoliating 1% (w/w GO:canola protein) GO prepared at various oxidation times (0.5 h:CPA GO-A, 2 h:CPA GO-B, 4 h:CPA GO-C). All adhesive samples were prepared in triplicate (*n* = 3) and minimum 5 wood samples per replicate were used for each strength measurement. Adhesion data was analyzed using one-way ANOVA followed by Duncan test for mean separation. Different letters on the bar represent significantly different adhesion strength (*p* < *0.05*). Error bars represent standard deviation.

Adhesives failure can happen in two occasions, either adhesively at adhesive-wood interface or cohesively within bulk adhesive material^28^. Since amorphous polymer generally has a limited mechanical strength^28^, cohesive failure is more prominent in biobased adhesives. The potential of nanomaterials in improving the bulk adhesion strength of canola protein adhesive was studied. Effects of adding GO on adhesion strength are shown in Fig. 1. All GO samples used in this study significantly increased (*p* < *0.05*) the adhesion strength and water resistance compared to the negative control and the pH control samples. GO prepared at 0.5, 2, and 4 h of oxidation showed a dry adhesion strength of 10.63 ± 0.81, 11.67 ± 1.00, and 11.22 ± 0.82 MPa, respectively.

Increasing oxidation time reduced the C/O ratio of GO samples, but showed an increasing trend in dry adhesion strength. Similar trend was also observed in soaked strength, where the highest strength of 10.73 ± 0.45 MPa was observed in GO-B (2 h of oxidation) followed by GO-C and GO-A samples (10.22 ± 0.45, 9.82 ± 0.38 MPa respectively). The wet adhesion was significantly increased from 1.98 ± 0.22 MPa in the pH control sample to 4.85 ± 0.35, 4.85 ± 0.61 and 4.48 ± 0.28 MPa for GO-A, GO-B and GO-C samples respectively, but did not differ among different oxidation times. Protein contains both hydrophilic and hydrophobic residues which makes it an excellent amphiphilic biopolymer with well-known adhesiveness to various solid surfaces^30^. Liu *et al*. (2010) studied the interactions of GO with bovine serum albumin (BSA) and suggested that conjugated GO-protein complex can act as an adhesive matrix to interact with other solid materials^30^. Studies on PVA polymer composites showed improved interactions and mechanical strength after exfoliating graphene oxide at low concentrations^28, 31^. Therefore, GO induced cohesive (protein-protein) and adhesive (protein-wood surface) interactions might be the main contributor to increased adhesion and water resistance observed in this study. Conversion of GO into more hydrophobic and stable reduced graphene oxide (rGO) might be another reason for the improved water resistance. Several authors reported thermal^32^ or protein aided reduction^30^ of GO into rGO in composite research, which improved the mechanical properties. Adhesive curing at elevated temperature, and the presence of canola protein might trigger the reduction of GO into rGO, thereby improve water resistance of the CPA-GO adhesive.

In comparison, canola protein modified with sodium bisulfite showed dry, wet and soaked adhesion strength of 5.28 ± 0.47, 4.07 ± 0.16, and 5.43 ± 0.28 MPa, respectively^6^. In another study, modifying canola protein with 0.5% sodium dodecyl sulphate (SDS) had dry, wet and soaked adhesion of 6.00 ± 0.69, 3.52 ± 0.48, and 6.66 ± 0.07 MPa, respectively. Grafting poly(glycidyl methacrylate) in canola protein was reported to improve the dry, wet and soaked adhesion to 8.25 ± 0.12, 3.80 ± 0.15, and 7.10 ± 0.10 MPa, respectively. Canola protein adhesives prepared with GO as developed in this study substantially improved both adhesion strength and water resistance.

### Changes in elemental composition, functional groups of GO and their effect on adhesion

**Figure 2:**
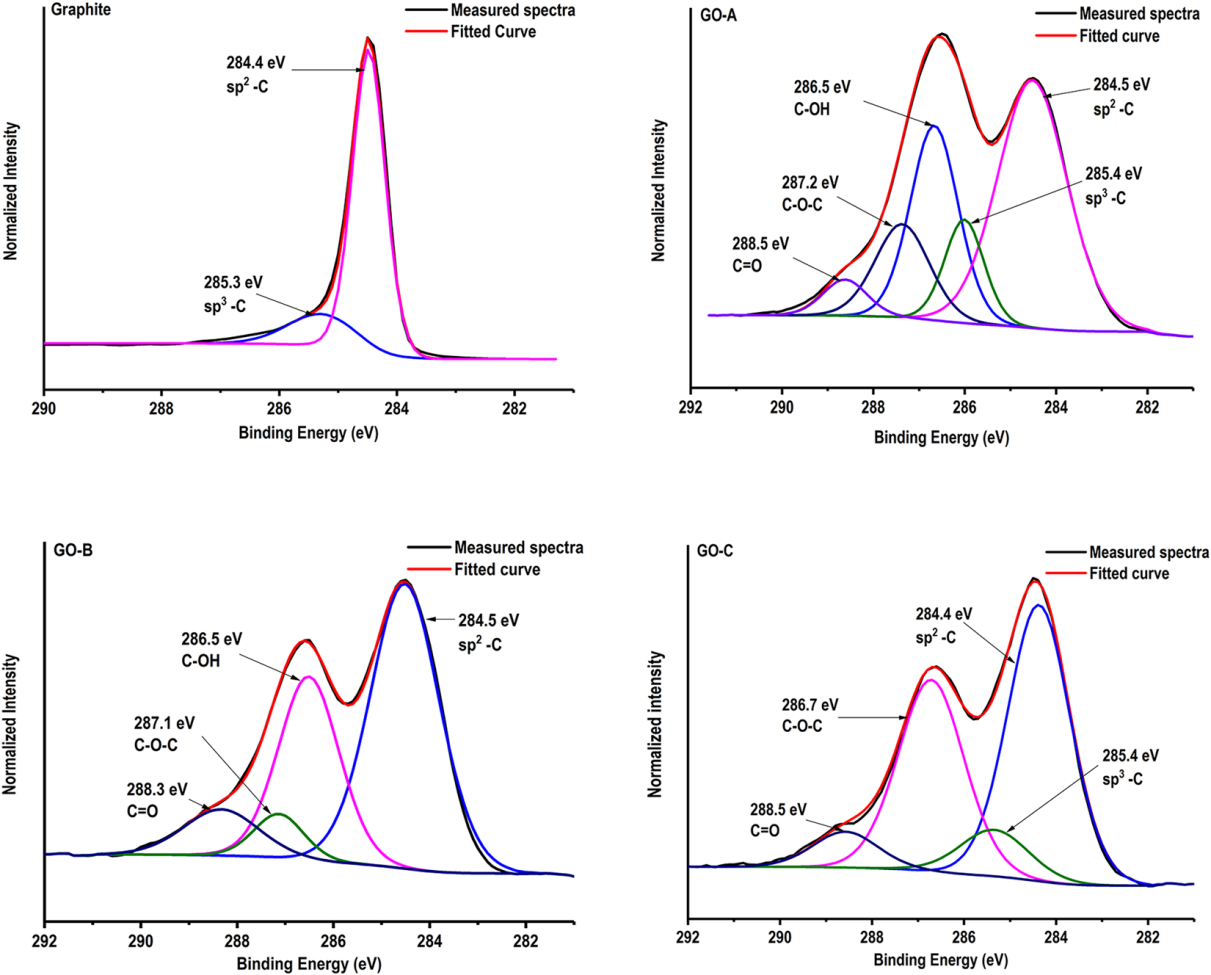
High-resolution carbon C 1 s scans of graphite and GO prepared with different oxidation times (Graphite–Without oxidation, GO-A–0.5 h, GO-B–2 h, and GO-C–4 h oxidation) obtained from X-ray photoelectron spectroscopy, and peak fitting showing oxidation time dependent changes in surface functional groups after graphite oxidation.

GO with variable elemental composition, C/O ratio and functional groups were previously developed via manipulating oxidation conditions^9, 23, 33^. In this study, we prepared GO with variable properties by changing oxidation time while maintaining other conditions constant. Oxidation conditions used in this study, elemental composition and C/O ratio of prepared GO samples are shown in Table 1. Native graphite mainly consists of carbon and oxygen at percentages of 97.65% and 2.35%, respectively, according to the XPS data (Supplementary information–S1). Graphite showed a C/O ratio of 41.55 while oxidizing graphite for 0.5, 2 and 4 h reduced C/O ratio to 2.06, 1.40 and 1.49, respectively. In addition, GO also contains small amount of sulfur (∼2%) and trace amounts of sodium, and manganese, as the residuals from GO processing. The presence of oxygen containing functional groups was confirmed by analyzing XPS high-resolution C1s spectra of graphite and GO samples. The original high-resolution C1s spectra and fitted peaks are shown in Fig. 2. XPS data processing for C1s spectra of graphite only showed a major peak centered at 284.5 eV which is attributed to sp^2^ hybridized carbon, derived from C = C and C-C bonds with delocalized π electrons^29, 33^. The other small peak at a binding energy of 285.3 eV resembles to sp^3^ carbon hybridization^34^, which attributed to oxidation of graphite in the presence of atmospheric oxygen^35^.

**Table 1:** Conditions used for oxidation of graphite and their effect on C/O ratio and elemental composition of prepared GO samples

Sample	Oxidation time	Oxidizer Amount (NaNO_3_)	C %	O%	S%	C/O ratio
Graphite	—	—	97.65	2.35	—	41.55
GO-A	0.5 h	5 g	65.60	31.85	2.54	2.06
GO-B	2 h	5 g	57.37	40.81	1.81	1.40
GO-C	4 h	5 g	57.06	38.20	2.32	1.49

GO-A sample shows four new peaks at binding energies around 285.5–288.5 eV, representing oxygen functional groups in addition to the characteristic sp^2^ peak at 284.5 eV. Shift of binding energies from 284.5 eV to 285.4 eV, 286.5 eV, 287.2 eV, and 288.5 eV are attributed to the occurrence of carbon sp^3^, C-OH, C-O-C, and C = O functional groups respectively. Previous studies reported similar binding energy shift in GO^36–38^. Increasing oxidation time to 2 h (GO-B sample) further changed the composition of surface functional groups. Peak corresponding to the carbon sp^3^ was disappeared while the relative proportion of C–OH and C = O peaks (286.5 eV and 288.3 eV respectively) increased. Furthermore, C–O–C peak appeared at the binding energy of 287.1 eV. Wang *et al*. (2012) also reported an increased proportion of C = O and C-OH groups at higher oxidation conditions in graphite oxide^29^. Further oxidation of graphite up to 4 h in GO-C increased the relative proportion of carbon sp^2^, C-O-C, and C = O groups, at the expense of C-OH groups; interestingly, the carbon sp^3^ peak re-appeared at 285.4 eV binding energy. Degradation of oxygen functional groups in prolonged oxidation might be the reason for sp^3^ hybridization observed in GO-C sample^33^.

**Figure 3:**
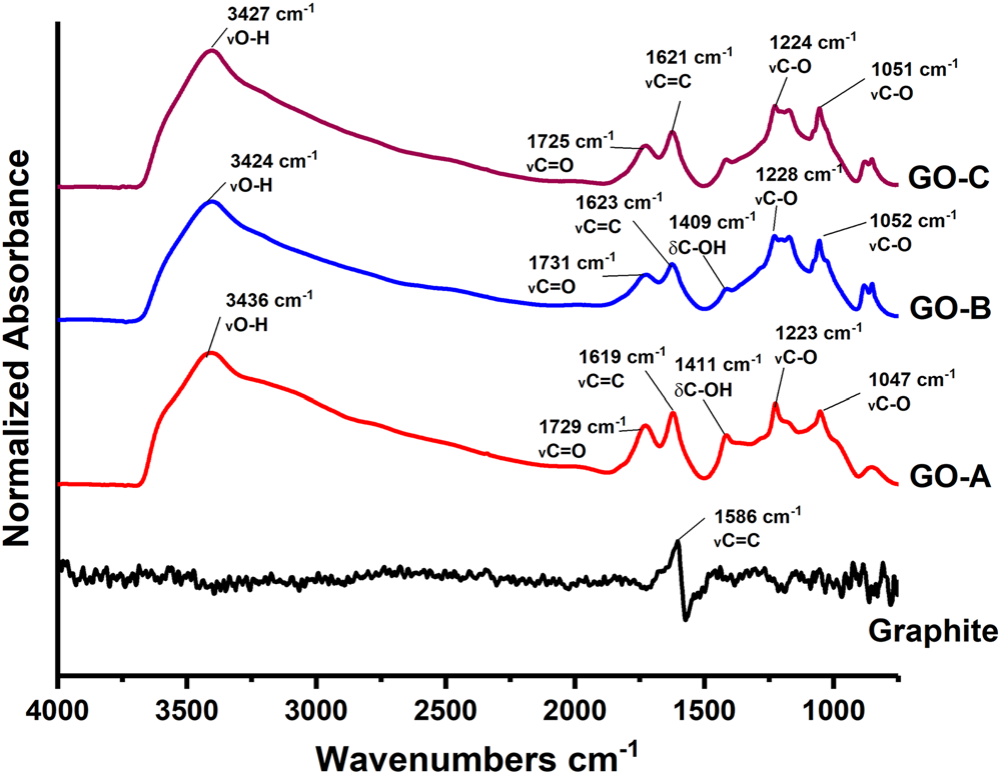
FTIR spectra of graphite and GO (graphite – Without oxidation, GO-A:0.5 h, GO-B:2 h, and GO-C:4 h oxidation) samples prepared with variable oxidation times showing oxidation dependent changes in GO functional groups.

FTIR spectra of GO samples prepared under different oxidation times are shown in Fig. 3. FTIR peaks were assigned to respective functional groups according to the previous data reported in the literature. In graphite, the peak appearing at 1586 cm^−1^ generally represents the stretching vibration of C = C bond (*v*C = C)^35, 39, 40^. However; after oxidation, the C = C stretching vibrations shifted to 1619 cm^−1^, 1623 cm^−1^, and 1621 cm^−1^ wavelengths for GO-A, GO-B and GO-C respectively. Chen *et al*. (2010), and Stankovich *et al*. (2006) also reported similar peak shifts in the range of 1618 cm^−1^–1626 cm^−1^ probably due to the oxygen functional groups present in GO^41, 42^. The absorption peaks of GO samples at 3424 cm^−1^–3436 cm^−1^ are attributed to the stretching vibration of –OH groups (*v*O-H) either from –OH groups of absorbed water or –OH groups formed during the oxidation^35, 41, 43^.

Following oxidation, the presence of new peaks at 1729 cm^−1^, 1731 cm^−1^, and 1725 cm^−1^ wavelengths respectively in GO-A, GO-B, and GO-C samples was observed; probably due to the formation of oxygen containing functional groups, causing the C = O stretching vibrations (*v*C = O)^29, 41, 42^. The intensity of *v*C = O in GO samples was increasing at increasing oxidation level. Wang *et al*. (2012) also reported similar trend at increasing oxidation levels^29^. Higher degree of oxidation and oxidation induced cracks in GO edges were reported as the main reasons for increased intensity of *v*C = O^29, 44, 45^. C-OH bending vibration (*δ*C-OH) peaks were observed in both GO-A and GO-B samples at 1411 cm^−1^ and 1423 cm^−1^ respectively, however the intensity was reduced in GO-C. Vibrations from either alcohols or carboxylic groups of GO were reported as the main contributors to *δ*C-OH^39, 40^. The peaks appeared at 1220 cm^−1^–1230 cm^−1^ range were usually assigned to C-O stretching vibrations (*v*C-O^35, 39–41^, which attributed to carboxylic acid groups^29^, hydroxyl groups^39, 41^, or epoxy groups^40, 46^ present in GO. The peaks appeared at 1730 cm^−1^–1731 cm^−1^ range were probably attributed to the ester groups that formed during graphite oxidation^47^.

The formation of various oxygen containing functional groups in GO might be responsible for the improved adhesion strength. For example, −OH groups in GO might increase −H bonding between adhesive matrix and wood surface; the epoxy groups (C-O-C) in GO can either homopolymerize with another epoxy group in GO, or react with functional groups such as −OH, −COOH on the wood surface, and −NH_2_, −SH in canola protein^48^, thus improving both adhesive and cohesive strength.

### Effect of different GO samples on protein structural changes

**Figure 4:**
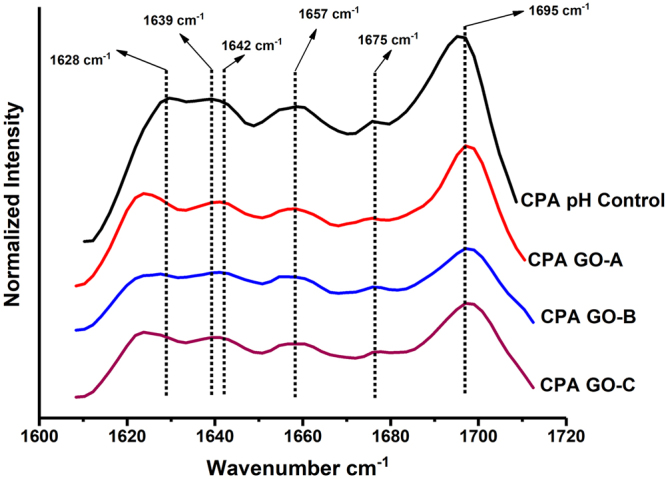
FTIR second derivative spectra showing changes in protein secondary structure of CPA-GO adhesives prepared by exfoliating GO (1% w/w GO:canola protein) with different oxidation levels. CPA pH Control: 10% w/v canola protein adhesive at pH 12.0; CPA GO-A: 10% w/v canola protein adhesive with 1% w/w (GO/protein) GO-A, CPA GO-B: 10% w/v canola protein adhesive with 1% w/w (GO/protein) GO-B, and CPA GO-C: 10% w/v canola protein adhesive with 1% w/w (GO/protein) GO-C.

Effect of different GO samples on secondary structure of canola protein was studied by creating second derivative of FTIR spectra followed by peak fitting of Amide I peak^49, 50^. GO induced protein secondary structural changes are shown in Fig. 4.

Exfoliating GO in canola protein has increased the relative proportions of unordered structures (1639–1642 cm^−1^ wavelength) and turn structures (at wavelength range of 1694–1697 cm^−1^) at the expense of β-sheets in the wavelengths of 1625 cm^−1^, 1636 cm^−1^and 1673–1675 cm^−1^ ^49, 50^. In comparison, GO-B and GO-C samples showed the highest relative proportions of unordered structures and turn structures, compared to the pH control and GO-A samples (Supplementary information–S2). The results observed in protein structural changes were compliment to the changes in adhesion strength of CPA-GO prepared with different GO samples. Increase in unordered structures will exposes more hydrophobic functional groups buried inside protein molecules which increase the hydrophobic interactions with wood surface^51^, thereby increase the water resistance and adhesion strength.

### Changes in GO crystallinity and their effect on GO dispersion in protein matrix

**Figure 5:**
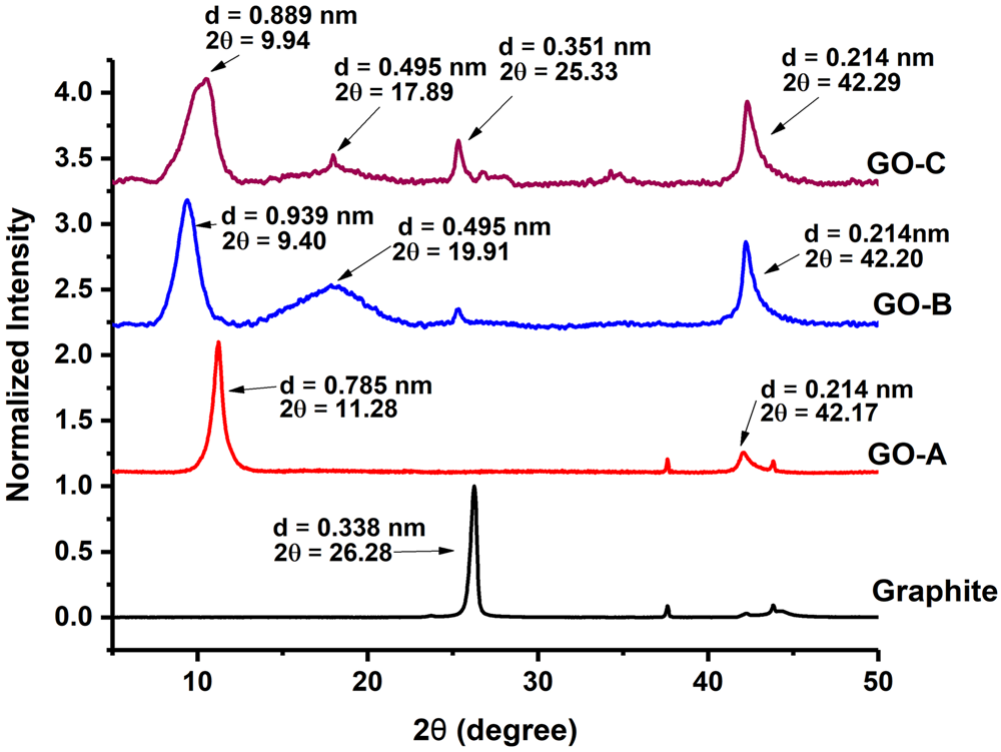
X-ray diffraction patterns, changes in glancing angle and interlayer spacing (calculated according to the Bragg’s equation: Sin θ = nλ/2d) of graphite and graphite oxide samples prepared with different oxidation times.

The effect of oxidation time on glancing angle (2θ) and interlayer spacing (*d*) of GO samples are shown in Fig. 5. X-ray diffraction of graphite showed one major crystalline peak at a glancing angle of 26.28° with *d* spacing of 0.338 nm. Shao *et al*. (2012) also reported a similar peak for graphite at a glancing angle of 26.54° and *d* spacing of 0.334 nm^23^.

After oxidation, the graphite crystalline peak was disappeared in GO-A (0.5 h) but two new peaks appeared at different glancing angles: the first major peak was appeared at glancing angle of 11.28° with *d* spacing of 0.785 nm while another minor peak was observed at glancing angle of 42.17° with *d* spacing of 0.214 nm. Shao *et al*. (2012) also reported the disappearance of the characteristic graphitic peak after oxidation and the formation of a new peak at a glancing angle of 11.3° with increased interlayer spacing of 0.80 nm^23^. Increasing graphite oxidation time from 0.5 h to 2 h significantly changed the crystallinity and *d* spacing of GO-B sample. Glancing angle of the characteristic GO peak has shifted from 11.28° to 9.40° while *d* spacing increased from 0.785 nm to 0.939 nm (for GO-A and GO-B respectively). Similar to GO-A, GO-B sample showed another peak at a glancing angle of 42.20° (*d* = 0.214 nm), and a new crystalline peak at 19.91° (*d* = 0.495 nm). Further increasing oxidation time to 4 h slightly shifted the glancing angle towards 9.94° while decreased *d* spacing to 0.889 nm.

The reduction in interlayer spacing has been previously reported due to the decomposition of oxygen containing functional groups in GO samples at prolonged oxidation^33, 52^. In GO-C, another two peaks were visible at glancing angles of 42.29°, and 17.89° with *d* spacing of 0.214 nm and 0.495 nm respectively. In addition, the new peak at a glancing angle of 25.33° (*d* = 0.351 nm) in GO-C showed similarity to the characteristic graphite peak appeared in un-oxidized graphite. The re-appearance of graphite like crystalline peak at higher oxidation level indicate the decomposition of oxygen containing functional groups, re-forming carbon sp^2^ bonds and reduction in crystallinity of GO-C samples^33, 52^.

**Figure 6:**
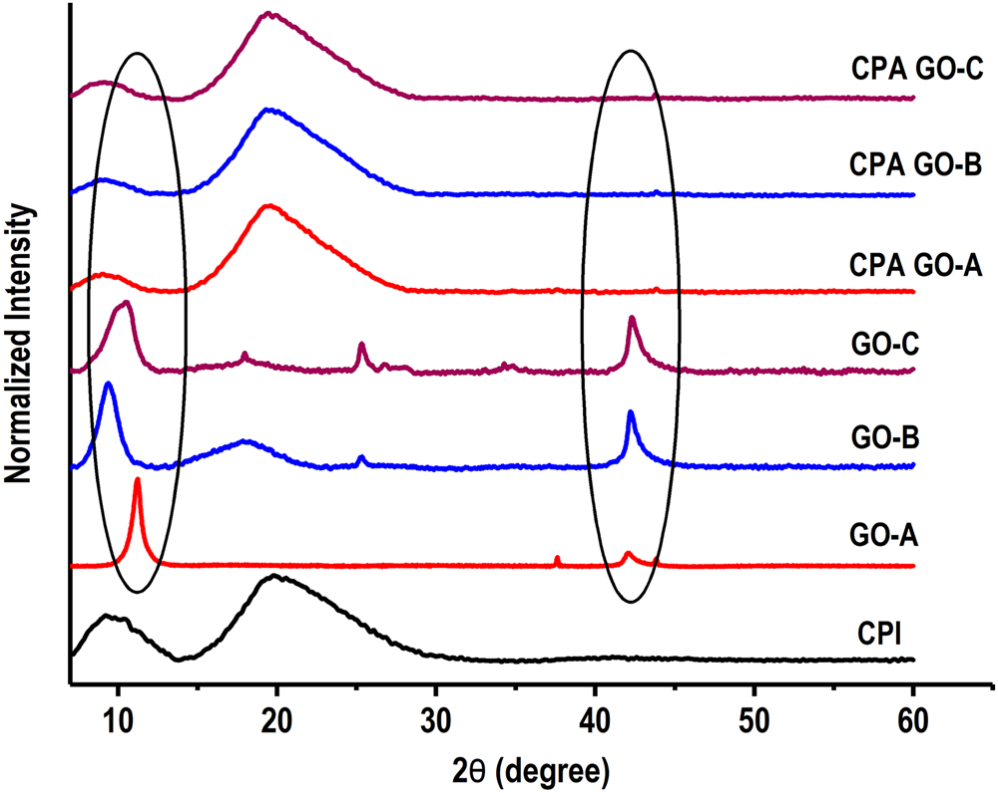
X-ray diffraction data showing the crystallinity of prepared graphite oxide samples under different oxidation time, and exfoliation properties of GO in canola protein matrix after adhesive preparation. CPI–Canola protein isolate, GO-A, GO-B and GO-C–starting graphite oxide samples prepared by oxidizing graphite for 0.5 h, 2 h, and 4 h respectively. CPA GO-A, CPA GO-B and CPA GO-C are prepared by exfoliating 1% w/w (GO/protein) GO-A, GO-B and GO-C respectively in 10% w/v canola protein dispersion.

Proper exfoliation of GO in polymer matrix is one of the major factors affecting the improvement of adhesion strength and water resistance. Aggregation of nanomaterial upon mixing with protein will not improve the adhesion strength^2, 53^; therefore it is important to produce GO with appropriate exfoliation properties. All three GO samples prepared in this study exhibit improved exfoliation in canola protein matrix. X-ray diffraction patterns of GO samples and their dispersion in canola protein are shown in Fig. 6. Two common crystalline peaks were appeared in all three GO samples with diffraction angles (2θ value) around ∼9–11° and ∼42° and one additional crystalline peak was found at ∼25° diffraction angle for GO-C. The disappearance of crystalline peaks after exfoliation of GO in canola protein clearly indicated the uniform exfoliation of GO within protein matrix.

**Figure 7:**
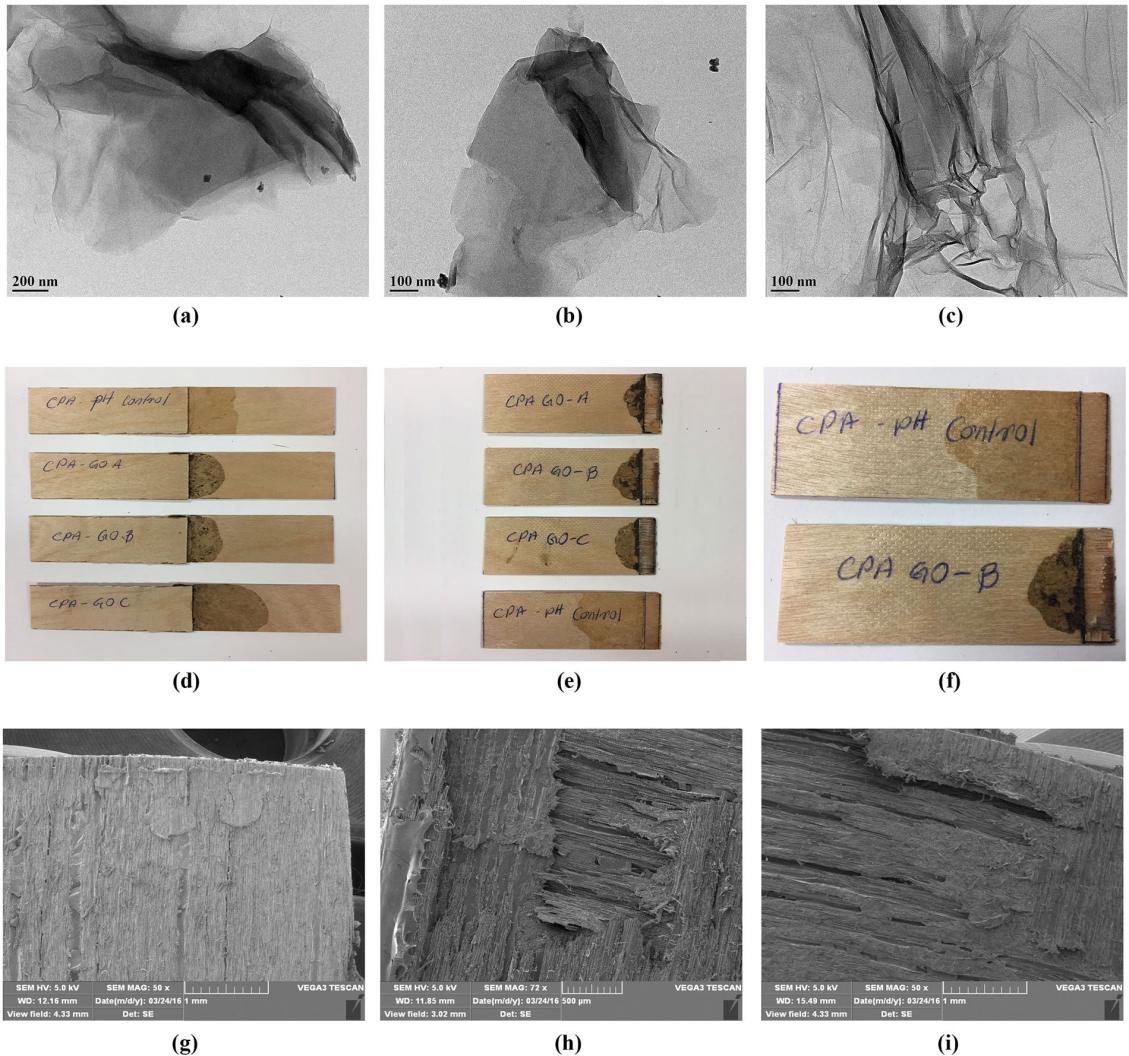
Image (a–c) represent the transmission electron microscopic (TEM) images of exfoliated graphite oxide samples prepared under different oxidation time in canola protein matrix. (a) CPA GO-A, (b) CPA GO-B and (c) CPA GO-C are prepared by exfoliating 1% w/w (GO/protein) GO-A, GO-B and GO-C respectively in 10% w/v canola protein dispersion. (d–f) represent the images of wood veneer samples bonded with CPA GO adhesives (d), and their fracture surface after strength testing (e,f). Images (g–i) represent the scanning electron microscopic (SEM) images of wood fracture surface after bond testing for (g) CPA GO-A adhesive, (h) CPA GO-B adhesive and (i) CPA GO-C adhesive

As shown in TEM images of exfoliated GO samples (Fig. 7), the appearance of single GO sheets in both CPA GO-A and CPA GO-B adhesive samples further supported the uniform exfoliation of GO in canola protein matrix. However, a slight aggregation of GO was visible in CPA GO-C. Addition of hydrophilic functional groups during graphite oxidation is the major reason for increased interlayer spacing of GO^33^. It was reported that increased interlayer space reduces binding energies of GO, which would facilitate the exfoliation of GO layers in the matrix^54^. Therefore, the uniform exfoliation of GO observed in this study, in particular for GO-B might be due to reduced binding energy resultant from increased interlayer spacing. Ultimately, proper exfoliation of GO will help in improving both adhesion strength and water resistance of the CPA-GO adhesive.

### Change in thermal properties of graphite oxide and their effect on thermal stability of prepared adhesive

**Figure 8:**
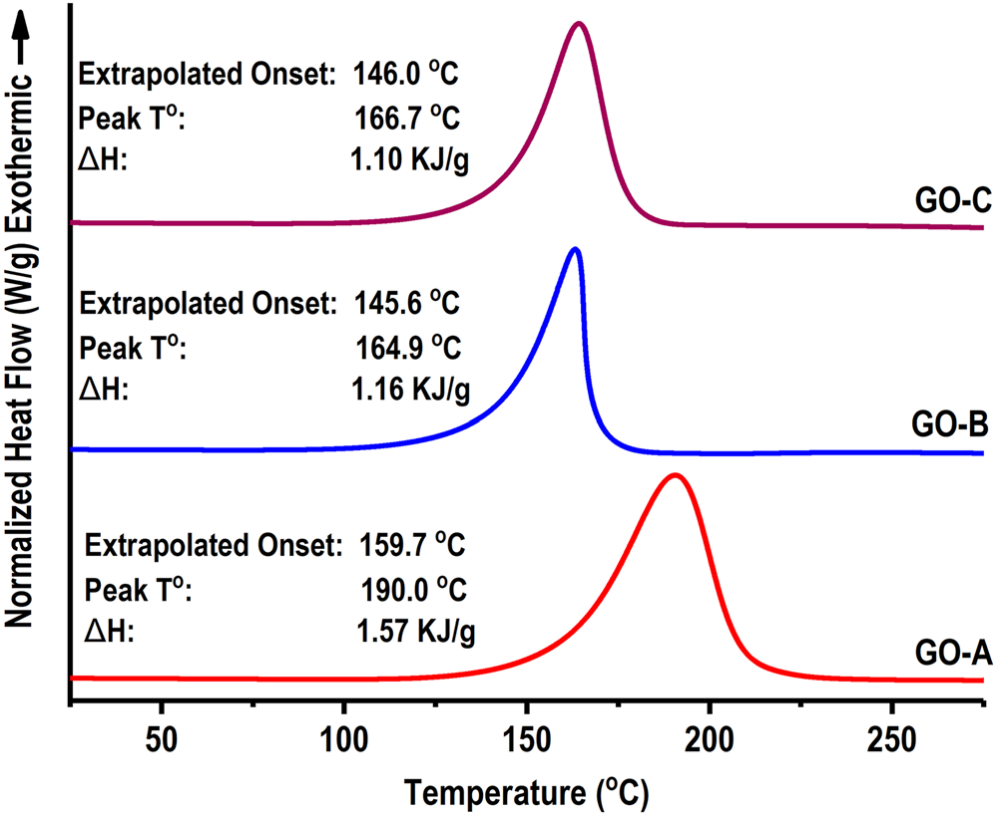
Changes in thermal properties of different graphite oxide samples prepared under different oxidation times (0.5 h: GO-A, 2 h: GO-B, 4 h: GO-C).

Effect of graphite oxidation time on GO thermal transitions is shown in Fig. 8. An exothermic transition was observed in all GO samples, but with different enthalpy requirement and temperature range. In GO-A (0.5 h) exothermic transition was observed at extrapolated onset and peak temperatures of 159.7 °C 190.0 °C respectively with 1.57 KJ/g ΔH. Increasing oxidation time to 2 h (GO-B) has changed the thermal transition to 145.6 °C, 164.9 °C and 1.16 KJ/g for extrapolated onset, peak temperature and ΔH respectively. Increasing oxidation time to 4 h (GO-C) shifted extrapolated onset and peak temperatures to 146.0 °C and 166.7 C° respectively where ΔH changed to 1.10 KJ/g. The reduction in ΔH and transition temperatures is a result of increased amount of oxygen containing functional groups. Schniepp *et al*. (2006) also reported similar changes in thermal transitions around ∼200 °C in graphite oxide and attributed them to decomposition of oxygen containing functional groups^36^. They have further analyzed the outlet gas generated from DSC, and showed that major products as CO_2_ and H_2_O that were generated during decomposition of oxygen containing functional groups^36^.

Effect of different GO samples on thermal transitions of CPA-GO are shown in Table 2. Adding GO into canola protein increased both onset and peak temperatures, as well as the specific heat in transitions. The increased thermal stability is an essential property for adhesive application, as it required to process under higher temperature for adhesive curing^28^. Adding nanomaterials, especially graphene oxide, have been proven to increase thermal stability of protein in previous studies mainly due to improved protein-protein/protein-GO interactions, and inherent thermal properties of GO. Linse *et al*. (2007) also reported an increased thermal stability and denaturation temperatures of soybean peroxidase enzyme conjugated with graphene oxide nanosheets^55^. Addition of GO into canola protein increased the thermal stability of all CPA-GO samples compared to control samples, which can be related to  the increased protein-protein/protein-GO interactions. CPA GO-A showed slightly higher onset and peak temperatures than that of CPA GO-B and CPA GO-C which can be a result of GO induced protein structural changes. Increased unordered structures were observed after adding GO-B and GO-C, at the expense of β-sheets and α-helix which can potentially reduce the thermal stability compared to GO-A.

**Table 2:** Effect GO exfoliation on thermal transitions of canola protein-GO hybrid wood adhesives (CPA GO). CPA GO-A, CPA GO-B and CPA GO-C are prepared by exfoliating 1% w/w (GO/protein) GO-A, GO-B and GO-C respectively in 10% w/v canola protein dispersion.

Adhesive Sample	Onset T° (°C)	Midpoint T° (°C)	Specific heat (J/g.C)
Canola Protein (−Control)	72.31 ± 1.86	89.43 ± 0.23	0.449 ± 0.07
Canola Protein (+Control)	85.14 ± 0.79	99.81 ± 2.01	1.202 ± 0.01
CPA GO-A	88.26 ± 1.67	105.64 ± 1.27	0.979 ± 0.13
CPA GO-B	83.44 ± 0.99	102.48 ± 1.83	1.260 ± 0.06
CPA GO-C	80.13 ± 1.94	99.25 ± 068	1.199 ± 0.10

## Conclusions

GO samples with various C/O ratio and surface functional groups were prepared at different oxidation time. Oxidation of graphite for 0.5, 2 and 4 h reduced the C/O ratio of graphite from 41.55 to 2.06, 1.40, and 1.49, respectively. The relative proportion of C-OH and C = O groups as well as interlayer spacing of GO were increased at increasing oxidation time from 0.5 h to 2 h whereas both C-OH content and interlayer spacing were reduced at 4 h of oxidation. GO prepared with different oxidation times improved both adhesion strength and water resistance in all three GO samples; the dry, wet and soaked strength was increased from 6.38 ± 0.84 MPa, 1.98 ± 0.22 MPa, 5.65 ± 0.46 MPa in the pH control sample to 11.67 ± 1.00 MPa, 4.85 ± 0.61 MPa, and 10.73 ± 0.45 MPa, respectively for GO-B exfoliated  adhesive. The improved adhesive and water resistance in GO added canola adhesive was due to increased interlayer spacing, improved exfoliation properties, and increased adhesive and cohesive interactions (protein-protein, protein-GO and adhesive-wood surface), hydrophobic interactions and thermal stability. Graphite oxide, instead of graphene, as we proposed for the first time in the study, is easier to process and more cost-effective in preparing protein-based wood adhesives with significantly improved functionalities.

## Methods

### Materials and Chemicals

Canola meal was provided by Richardson Oilseed Ltd. (Lethbridge, AB, Canada). All chemicals were purchased from Fisher Scientific (Ottawa, ON, Canada) unless otherwise noted. Graphite and cellulose were purchase from Sigma-Aldrich (Sigma Chemical Co, St. Louise, MO, USA). Birch wood veneer with thickness of 0.7 mM was purchased from Windsor Plywood Co (Edmonton, AB, Canada).

### Canola protein extraction

Proteins were extracted from defatted canola meal as described by Manamperi *et al*. (2010) with slight modifications^56^. Meal was ground to a fine powder using a Hosokawa milling and classifying system (Hosokawa Micron Powder Systems, Summit, NJ, USA) and then passed through a 100-mesh size sieve. Ground canola meal was mixed with mili-Q water in 1:10 (w/v) ratio; pH was adjusted to 12.0 by adding 3 M NaOH and stirred for 30 m (300 RPM, room temperature). The resulting dispersion was centrifuged for 15 m (10000 g, 4 °C). The supernatant was collected, pH was readjusted to 4.0 by adding 3 M HCl, stirred for another 30 m, and centrifuged at the same condition above to collect protein precipitate. The precipitate was washed with deionized water, freeze-dried, and stored at −20 °C for further use.

### Graphite oxide preparation

Graphite oxide nanoparticles (GO) were prepared as described by Hummers and Offeman method^57^ with modification for oxidation time to produce GO with different oxidation levels. In brief, 5 g of graphite and 5 g of NaNO_3_ were mixed in a glass beaker and 120 mL of concentrated H_2_SO_4_ was slowly added while stirring in an ice bath at 200 RPM for 0.5 h, 2 h, and 4 h to prepare GO-A, GO-B and GO-C samples respectively. Then, 15 g of KMnO_4_ was slowly added to the reaction mixture while maintaining the temperature at 35 ± 3 °C with stirring for 1 h. At the end of the reaction, 92 mL of deionized water was added and stirred for 15 m. Unreacted KMnO_4_ and other leftover chemicals were neutralized by adding 80 mL of hot (60 °C) deionized water containing 3% H_2_O_2_. After cooling to room temperature, samples were centrifuged (10000 g, 15 m, 4 °C) and washed with deionized water to remove any leftover chemicals. Collected GO samples were sonicated for 5 m (at 50% power output); freeze dried, further dried in a vacuum desiccator with P_2_O_5_, and stored in air tight containers at -20 °C for further use.

### Preparation of canola protein-graphite oxide hybrid wood adhesive (CPA-GO)

GO with different C/O ratios was exfoliated in canola protein matrix according to our previously reported method. 1% (w/w, GO/protein) GO addition level was selected based on the optimum conditions developed in our previous method^8^. In brief, 3 g of canola protein was mixed with 20 mL of deionized water (15% w/v solution) and stirred for 6 h (300 rpm) at room temperature to disperse canola proteins; and then the pH was adjusted to 5.0 using 1 M HCl solution. GO samples (GO-A, GO-B and GO-C) were separately dispersed in 10 mL of deionized water (equivalent to a final GO/protein ratio of 1%, w/w, GO/protein) by stirring (300 rpm) 5 h at room temperature and another 1 h at 45 ± 3 °C, sonicated for 3 m by providing intermittent pulse dispersion of 5 s (at 3 s intervals and 60% amplitude) using medium size tapered tip attached to a high intensity ultrasonic dismembrator (Model 500, Thermo Fisher Scientific INC, Pittsburg, PA, USA), and then homogenized for 2 m (2000 rpm) using ULTRA TURRAX high shear homogenizer (Model T25 D S1, IKA^®^ Works, Wilmington, NC, USA). The prepared GO dispersions were slowly added to the protein dispersions dropwise while stirring for 15 m (300 rpm) to have a final protein concentration of 10% (w/v) in the adhesive mixture. The resulting adhesive mixtures were sonicated and homogenized as above and the pH of the adhesive was adjusted to 12.0 by adding 6 M NaOH solution. Negative control was prepared by dispersing canola protein (10% w/v) in deionized water and use as is while pH control was prepared by adjusting the pH of canola protein dispersions (10% w/v) to 12.0 similar to GO dispersed samples, without adding GO.

### Adhesion strength measurement

Hardwood veneer samples (Birch, 1.2 mm thickness) were cut into a dimension of 20 mm × 120 mm (width and length) using a cutting device (Adhesive Evaluation Systems, Corvallis, OR, USA). Veneer samples were conditioned for seven days at 23 °C and 50% humidity in a controlled environment chamber (ETS 5518, Glenside, PA, USA) according to the specifications of ASTM D2339-98 (2011) standard method^58^. CPA-GO hybrid adhesives were spread at an amount of 40 uL/veneer strand in a contact area of 20 mm × 5 mm using a micropipette. Veneer samples were air dried for 5 m and hot pressed for 10 m (at 120 °C and 3.5 MPa) using Carver manual hot press (Model 3851-0, Carver Inc., In, USA). Dry adhesion strength (DAS) was measured according to the ASTM standard method D2339-98 (2011) by measuring tensile loading required to pull bonded veneer using Instron machine (Model 5565, Instron, MA, USA) equipped with 5 kN load cell. Tensile strength data was collected using Bluhill 3.0 software (Instron, MA, USA). Wet adhesion strength (WAS) and soaked adhesion strength (SAS) was measured according to the ASTM standard method D1151-00 (2013)^59^ using instron tensile loading. WAS values were measured after submerging bonded veneer samples for 48 h in water (23 °C) where SAS was measured after reconditioning submerged veneer samples for seven days at 23 °C and 50% relative humidity in a controlled environment chamber (ETS 5518, Glenside, PA, USA). Minimum of four bonded veneer samples per formulation were used in testing strength (DAS, WAS, SAS). All samples were clamped to Instron with a 35 mm gauge length and tested at 10 mm/m cross head speed.

### X-ray Photoelectron spectroscopy (XPS)

GO samples were characterized using X-ray photoelectron spectroscopy (XPS) for their elemental composition, carbon/oxygen (C/O) ratio and changes in the functional groups according to their oxidation time. Samples were analyzed using monochromatic Al K α radiation (1486.6 eV) generated from Kratos Axis 165 X-ray spectrometer (Kratos Analytical Ltd. UK). Resulting spectra’s were analyzed by CasaXPS software V2.3.16 PR 1.6 (Casa Software Ltd) for elemental composition and C/O ratio. Binding energy of neutral carbon C1s spectra was adjusted to 284.5 eV as a reference. Oxidation time dependent changes in surface functional groups were characterized by curve fitting of high-resolution C1s spectra assuming a Shirley background and 70%/30% Gaussian/Lorentzian distribution shape. Four peaks were fitted for all other GO samples while five peaks were used in GO-A sample with a lower oxidation time.

### X-ray diffraction (XRD)

X-ray diffraction (XRD) of GO and CPA-GO samples were performed using Rigaku Ultima IV powder diffractometer (Rigaku Co. Japan). Cu-Kα radiation (0.154 nm) was used to collect angle data (2ϴ) from 5 to 50 degrees. Interlayer spacing of graphite oxide was calculated using Bragg’s equation^60^; sin θ = nλ/2d where, λ, d and θ represent wavelength of the radiation, spacing between diffraction lattice (interlayer space), and glancing angle (measured diffraction angle) respectively^53, 61^. XRD data was analyzed using Origin 2016 software (OriginLab Corporation, MA, USA) to identify effect of oxidation time on exfoliation of GO.

### Differential scanning calorimetry (DSC)

Thermal transitions of GO and CPA-GO adhesives were studied using differential scanning calorimeter (Perkin-Elmer, Norwalk, CT, USA). DSC instrument was calibrated for temperature and heat flow using a pure indium reference sample. Sample moisture was first removed by freeze-drying followed by drying with P_2_O_5_ for two weeks in a hermetically sealed desiccator. GO and hybrid adhesive samples were accurately weighed into T-Zero hermetic aluminum pans (∼6 mg each), mixed with 60 µL of 0.01 M phosphate buffer, and hermetically sealed with lids. Heat flow differential of samples were recorded against the empty reference pan under continuous nitrogen purging. All samples were equilibrated at 0 °C for 10 m and thermodynamic data was collected while heating from 0 to 250 °C at a ramping rate of 10 °C m^−1^. Data was analyzed using Universal Analysis 2000 software for thermal transition changes in adhesives and GO samples (Perkin-Elmer, Norwalk, CT, USA).

### Fourier transform infrared spectroscopy (FTIR)

Effect of oxidation time on GO functional groups and GO induced protein secondary structural changes in adhesive samples were characterized using Nicolet 8700 Fourier transform infrared spectrometer (Thermo Eletron Co. WI, USA). Sample moisture was removed prior to FTIR analysis by freeze-drying and further drying with P_2_O_5_ in a hermetic desiccator for two weeks. Samples were mixed with potassium bromide (KBr), milled into a powdered pellet prior to FTIR analysis. IR spectra were collected in 400–4000 cm^−1^ range using 128 scans at a resolution of 4 cm^−1^. Collected data was analyzed using Origin 2016 software (OriginLab Corporation, MA, USA) to identify changes in functional groups. Second derivative spectra were generated using Savitzky-Golay smooth function (7 points window) and used for curve fitting to identify GO induced protein structural changes.

### Transmission Electron Microscopy (TEM)

Effect of GO samples on exfoliation in canola protein matrix were characterized using transmission electron microscopy (TEM). Images were collected using Philips/FPI transmission electron microscope (Model Morgagni, FEI Co, OR, USA) coupled with Getan digital camera (Getan Inc, CA, USA). Adhesive samples were diluted to 100-fold with ethanol, and a single drop was casted onto 200 mesh holey copper grid covered with carbon film. After 30 seconds of air-drying, the remaining liquid was removed and copper grid was used for collecting TEM images.

### Statistical Analysis

Adhesion strength data (DAS, WAS, and SAS) was analyzed using analysis of variance (ANOVA) followed by Duncan’s Multiple Range (DMR) test to identify the effects of graphite oxidation time on adhesion strength. Collected data was processed using Statistical Analysis System Software (SAS version 9.4, SAS Institute, Cary, NC). Effects of different GO samples on adhesion strength were evaluated at the 95% confidence level.

### Data Availability

All data generated or analysed during this study are included in this published article (and its Supplementary Information files).

## Electronic supplementary material

Supplementary Information
