# Supplementary material for: Graphite Oxide Improves Adhesion and Water Resistance of Canola Protein–Graphite Oxide Hybrid Adhesive
Source: Sci Rep. 2017 Sep 14;7:11538. doi: 10.1038/s41598-017-11966-8 (PMC5599558; doi:10.1038/s41598-017-11966-8)
Supplement: Supplementary file 1 — Supplementary Information [file 41598_2017_11966_MOESM1_ESM.doc]

***Electronic Supplementary Information***

Graphite Oxide Improves Adhesion and Water Resistance of Canola Protein–Graphite Oxide Hybrid Wood Adhesive

***Nandika Bandara, Yussef Esparza, Jianping Wu*,***

Department of Agricultural, Food and Nutritional Science, University of Alberta, Edmonton, Canada

*Corresponding author:

Tel: (1-780)492-6885 Fax: (1-780)492-4265

E-mail: [jwu3@ualberta.ca](mailto:jwu3@ualberta.ca)

**Figure S1 - Elemental composition and C/O ratio of prepared graphite oxide samples**

*Figure S1: a – X-ray photoelectron spectra showing elemental composition and C/O ratio in un-oxidized graphite*

*Figure S1: b – X-ray photoelectron spectra showing elemental composition and C/O ratio in graphite oxide prepared with 0.5 hrs oxidation time (GO-A sample)*

*Figure S1: c – X-ray photoelectron spectra showing elemental composition and C/O ratio in graphite oxide prepared with 2 hrs oxidation time (GO-B sample)*

*Figure S1: d – X-ray photoelectron spectra showing elemental composition and C/O ratio in graphite oxide prepared with 4 hrs oxidation time (GO-C sample)*

**Table S1**. Relative amount (%) of each functional group present in graphite and graphite oxide samples.

|  | **Area of fit peak, % (FWHM, eV)** | | | | |
| --- | --- | --- | --- | --- | --- |
|  | sp^2^ | sp^3^ | C-OH | C-O-C | C=O |
| **Graphite** | 82.30 (0.66) | 17.69 (1.27) | - | - | - |
| **GO-A** | 43.43 (1.71) | 10.93 (1.27) | 25.50 (1.23) | 15.32 (1.60) | 4.80 (1.33) |
| **GO-B** | 54.59 (1.66) | - | 28.66 (1.55) | 7.55 (1.30) | 9.20 (1.91) |
| **GO-C** | 51.22 (1.62) | 8.23 (1.73) | - | 34.11 (1.55) | 6.42 (1.59) |

Area percentages from XPS high resolution C1s curve fitting. FWHM is the full width at half maximum of fitted peaks.

**Figure S2: Secondary structural changes in adhesive samples**

*Figure S2:a – Peak fitting of FTIR second derivative spectra showing changes in protein secondary structure in pH Control adhesive sample*

*Figure S2:b – Peak fitting of FTIR second derivative spectra showing changes in protein secondary structure in CPA GO-A adhesive sample*

*Figure S2: c – Peak fitting of FTIR second derivative spectra showing changes in protein secondary structure in CPA GO-B adhesive sample*

*Figure S2: d – Peak fitting of FTIR second derivative spectra showing changes in protein secondary structure in CPA GO-C adhesive sample*
